# Development of Extragenital Lichen Sclerosus in Malignant Melanoma Patients Treated With Ipilimumab in Combination With Nivolumab

**DOI:** 10.3389/fonc.2020.573527

**Published:** 2020-10-07

**Authors:** S. Morteza Seyed Jafari, Laurence Feldmeyer, Robert E. Hunger

**Affiliations:** Department of Dermatology, Inselspital, Bern University Hospital, University of Bern, Bern, Switzerland

**Keywords:** checkpoint inhibitors, extragenital lichen sclerosus et atrophicus, ipilimumab, malignant melanoma, metastatic, nivolumab

## Abstract

The immune checkpoint inhibitors opened a new era for the treatment of melanoma. Nowadays, combined immune checkpoint inhibitors are administered to provide additive or synergistic effects on anti-melanoma immunity. The use of these drugs comes with serious adverse events related to excessive immune activation. Here, we present development of extragenital lichen sclerosus in a patient with metastatic malignant melanoma, during the combined therapy with checkpoint inhibitors.

## Introduction

The immune checkpoint inhibitors opened a new era for the treatment of melanoma (1). As potent immune checkpoint inhibitors, antibodies against cytotoxic T-lymphocyte-associated protein 4 (CTLA-4) and programmed cell death protein 1 (PD-1) are currently in clinical use worldwide (1). Ipilimumab, a human IgG1 monoclonal antibody, blocks CTLA-4 (1). Nivolumab, a fully human IgG4 anti-PD-1 antibody, blocks selectively the interaction of PD-1 and PD-L1/PD-L2 (1). Nowadays, combined immune checkpoint inhibitors are administered to provide additive or synergistic effects on anti-melanoma immunity (1). The use of these drugs comes with serious adverse events related to excessive immune activation, collectively known as immune-related adverse events, such as colitis, hepatitis, thyroiditis, lichenoid dermatoses, bullous dermatoses, psoriasis, and morphea (2, 3). The recent papers by Wernham et al. (3) and di Meo et al. (2) described an association between nivolumab treatment and genital and extragenital lichen sclerosus, respectively. However, there are no cases in the literature on the association between combined immune checkpoint inhibitors and extragenital lichen sclerosus. Here, we present development of extragenital lichen sclerosus in a patient with metastatic malignant melanoma, during the combined therapy with checkpoint inhibitors.

## Report of the Cases

**FIGURE 1:**
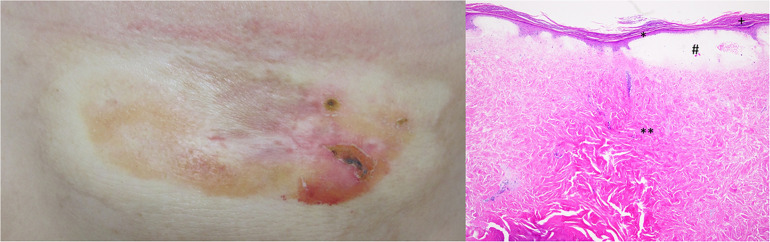
Clinical image shows an atrophic, indurated, and hypopigmented plaque on left *submammary* region. Histologically, extragenital lichen sclerosus is characterized by hyperkeratosis (+), epidermal atrophy with flattening of rete ridges (*), edema in the superficial dermis (#), and homogenized collagen in the dermis with subtotal loss of skin adnexal structures (**).

A primary melanoma with 0.6-mm Breslow depth and without ulceration was detected on the back of a 39-year-old female patient. Thereafter, the patient underwent regular clinical follow-up. Six years later, during a routine control, metastatic lesions in the brain and lung were diagnosed. Therefore, a combined therapy with ipilimumab and nivolumab was initially started and followed by monotherapy with nivolumab, as discussed before (4). The therapy was stopped after 2 years with acceptable clinical response. However, during the last months of the therapy, she presented with an itchy atrophic, indurated hypopigmented plaque on left submammary region (Figure 1). The clinical suspicion of extragenital lichen sclerosus was confirmed by histological evaluation of the lesion revealing hyperkeratosis, epidermal atrophy with flattening of rete ridges, edema in the superficial dermis, and homogenized collagen in the dermis with subtotal loss of skin adnexal structures (Figure 1). Therefore, a local therapy with clobetasol propionate 0.05% cream was initiated. After 2 weeks of daily application, the therapy was reduced to twice weekly, and a daily therapy with tacrolimus ointment 0.1% was added. This combination therapy significantly improved the patients’ itch and induration of the plaque.

## Discussion

To our knowledge, our case is the first report of an association between extragenital lichen sclerosus during the combined therapy with checkpoint inhibitors. Similar to the cases reported by Wernham et al. (3) and di Meo et al. (2), our patient presented with an itchy atrophic, indurated hypopigmented plaque during the therapy. The etiology of lichen sclerosus, a chronic inflammatory connective tissue disorder, has not been fully elucidated (3, 5–7). However, the increased prevalence of organ-specific autoantibodies in lichen sclerosus, such as extracellular matrix protein 1, supports that the autoimmune mechanisms might play a pathogenetic role (5, 6, 8). In addition, increased levels of Th1-specific cytokines, dense T-cell infiltrates, and enhanced BIC/miR-155 expression has been found (2, 3, 7). This might explain an association of lichen sclerosus with checkpoint inhibitor therapy. As ipilimumab increases activated T cells and enhances humoral immunity and nivolumab prevents the deactivation of T lymphocytes, thus promoting self-reactive T cells (2, 9), both drugs might lead to autoimmune diseases, such as lichen sclerosus, as reported before (2, 3).

In conclusion, extragenital lichen sclerosus should be considered as one of the immune-related adverse events of combined therapy with checkpoint inhibitors. Local therapy with corticosteroids and/or calcineurin inhibitors could reduce the patients’ discomfort and avoid disease progression. Despite presence of strong clinical and histological clues in the patients, occurrence of extragenital lichen sclerosus irrelevant to the immune therapy could not be excluded. Therefore, further studies might be demanded to investigate this issue in more details.

## Data Availability Statement

The raw data supporting the conclusions of this manuscript will be made available by the authors, without undue reservation, to any qualified researcher.

## Ethics Statement

Ethical review and approval was not required for the study on human participants in accordance with the local legislation and institutional requirements. The patients/participants provided their written informed consent to participate in this study.

## Author Contributions

SS, LF, and RH designed the study and performed acquisition, analysis, and interpretation of data, performed critical revision of the manuscript for important intellectual content. SS wrote the manuscript. All authors contributed to the article and approved the submitted version.

## Conflict of Interest

The authors declare that the research was conducted in the absence of any commercial or financial relationships that could be construed as a potential conflict of interest.
